# Left Ventricular Pseudoaneurysm: A Rare but Fatal Complication of Myocardial Infarction

**DOI:** 10.7759/cureus.51480

**Published:** 2024-01-01

**Authors:** Sehajpreet Singh, Jasveen Kaur, Arjun Basnet, Ravi Jayanti, Bilal A Malik

**Affiliations:** 1 Internal Medicine, Maimonides Medical Center, Brooklyn, USA; 2 Cardiology, Maimonides Medical Center, Brooklyn, USA

**Keywords:** myocardial rupture, left ventricular pseudoaneurysm, cardiology imaging, myocardial infarction, cardiac surgical procedures

## Abstract

Left ventricular pseudoaneurysm is a ventricular free wall rupture contained within the adjacent adherent pericardium or scar tissue. Myocardial infarction (MI), cardiac surgery, and chest trauma are the common causes. The most common presenting symptoms of pseudoaneurysms are congestive heart failure, chest pain, and dyspnea, but a small percentage of patients may be asymptomatic. Early diagnosis and treatment are of prime importance because of the tendency of pseudoaneurysms to expand and rupture, with a high mortality rate, especially if left untreated. We present a case of a 65-year-old man who was found to have left ventricular pseudoaneurysm on a follow-up echocardiography within three weeks of an MI. He subsequently underwent patch repair and was discharged after medical optimization. Our case highlights the importance of maintaining a high clinical suspicion of pseudoaneurysm in a patient post-MI, as delayed diagnosis and treatment can be fatal.

## Introduction

Left ventricular pseudoaneurysms develop as a complication of myocardial infarction. It is a ventricular free wall rupture that is contained by the surrounding pericardium or fibrous tissue with a narrow neck communicating with the left ventricle. They are differentiated from true aneurysms by the absence of endocardium or myocardium and a tendency to expand and rupture. The incidence of left ventricular pseudoaneurysm is extremely low, with an incidence rate of only 0.23% [1]. Here, we report a case of a 65-year-old man who presented with a post-myocardial infarction left ventricle pseudoaneurysm.

## Case presentation

**Figure 1:**
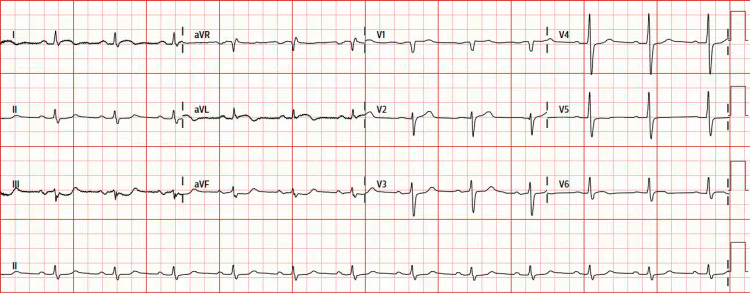
Electrocardiography (ECG) on arrival Electrocardiography (ECG) on arrival showed sinus rhythm with minimal ST-segment elevation in the lateral leads and minimal ST-segment depression in the inferior leads.

A 65-year-old man with a history of hypertension, diabetes mellitus, and prior smoking presented with non-radiating, substernal chest tightness for three days. Electrocardiography (ECG) on arrival showed sinus rhythm with minimal ST-segment depression in the inferior leads and minimal ST-segment elevation in the lateral leads (Figure 1).

**Figure 2:**
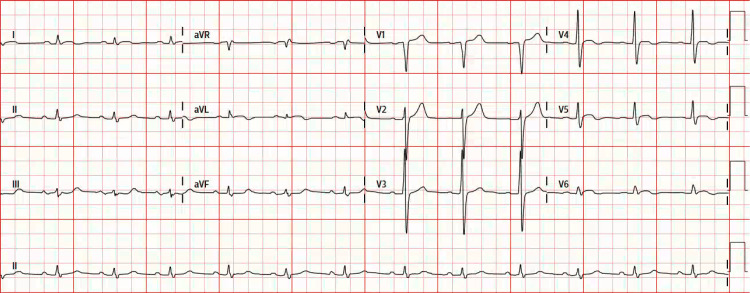
Repeat electrocardiography Repeat electrocardiography showed worsening ST-segment elevations in the lateral leads.

Repeat electrocardiography showed worsening ST-segment elevations in the lateral leads (Figure 2).

Cardiac troponin-I was 5.27. A 2-dimensional transthoracic echocardiography (2D-TTE) showed ejection fraction (EF) of 41-45% with multiple wall motion abnormalities in the apical, inferolateral, and anterolateral left ventricle (LV) (Videos 1-3).

**Video 1:** TTE parasternal (long axis view) of the heart TTE: Transthoracic echocardiography TTE parasternal (long axis view) of the heart shows hypokinesis of the inferior wall.

**Video 2:** TTE parasternal (short axis view) of the heart Transthoracic echocardiography (TTE) parasternal (short axis view) of the heart showing hypokinesis of the inferior and anterolateral walls.

**Video 3:** TTE (apical view) of the heart Transthoracic echocardiography (TTE, apical view) of the heart shows hypokinesis of the apex and lateral wall.

The urgent cardiac angiogram was performed, which showed 100% occlusion of Obtuse Marginal-1 (OM1), 90% occlusion of Diagonal-1 (D1), and 70% occlusion proximal Left Anterior Descending (pLAD) artery (Video 4).

**Video 4:** RAO (caudal view) of the coronary angiogram Right Anterior Oblique (RAO, caudal view) of the coronary angiogram showing 100% occlusion of Obtuse Marginal-1 (OM1), 90% occlusion of Diagonal-1 (D1), and 70% occlusion proximal Left Anterior Descending (pLAD) artery.

A stent was placed in OM1 (culprit vessel), and the patient was scheduled for a staged percutaneous coronary intervention (PCI) of the D1 and pLAD (Video 5).

**Video 5:** Anteroposterior view of the coronary angiogram after revascularization of the OM1

**Figure 3:**
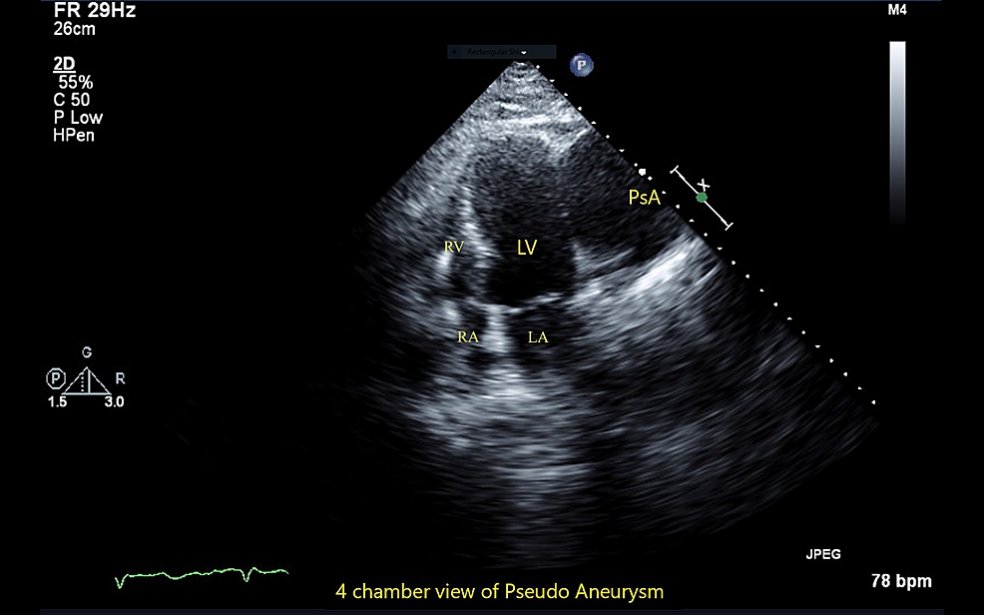
2D-TTE (apical four-chamber view) The image shows a thinned-out LV lateral wall and a contained LV free wall rupture with the formation of a large (8 cm) Pseudoaneurysm. 2D-TTE: Two-dimensional transthoracic echocardiography, LA: left atrium, LV: left ventricle, RA: right atrium, RV: right ventricle, PsA: pseudoaneurysm.

**Figure 4:**
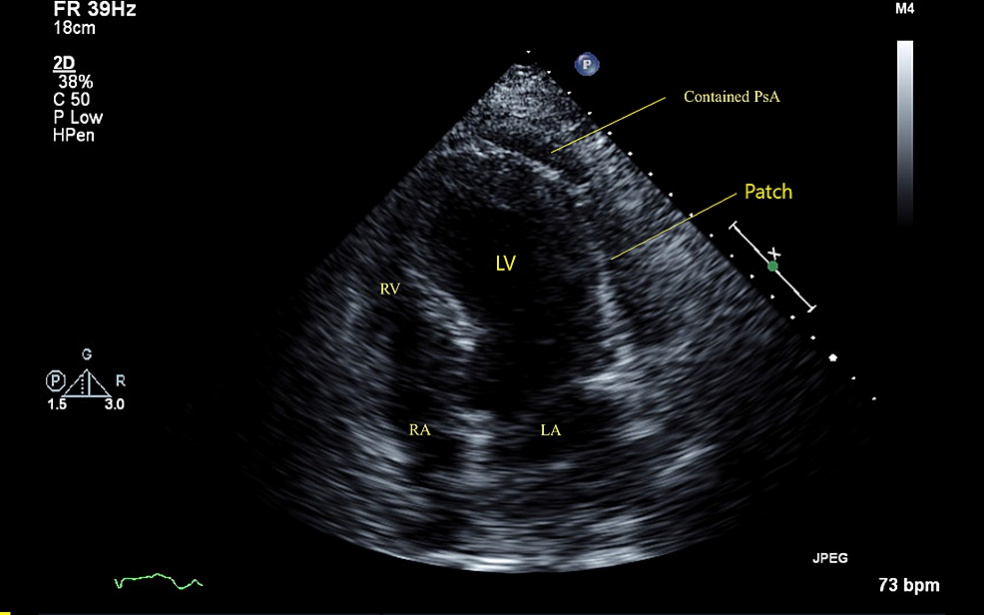
2D-TTE (apical four-chamber view) The image shows contained pseudoaneurysm of LV lateral wall consistent with Hemashield patch repair. 2D-TTE: Two-dimensional transthoracic echocardiography, LA: left atrium, LV: left ventricle, RA: right atrium, RV: right ventricle, PsA: Pseudoaneurysm

The patient later developed ventricular fibrillation cardiac arrest requiring an intra-aortic balloon pump (IABP) for cardiogenic shock, subsequently undergoing percutaneous coronary intervention with stent placement in pLAD and D1. The hospital course was further complicated by the development of pneumonia requiring intubation and the development of acute renal failure requiring dialysis. The patient was subsequently discharged after Implantable Cardioverter Defibrillator (ICD) placement with arrangements for follow-up. Upon follow-up after three weeks, the patient mentioned making progress while walking short distances, while still reporting baseline dyspnea on exertion. On examination, he was alert and oriented. Blood pressure was 140/80, pulse was 65 and temperature was 98.5°F. There was decreased air entry into the left lower lobe, first and second heart sounds were normal, and no murmurs were appreciated. There was no jugular venous distension or peripheral edema, and the remainder of the examination was unremarkable. Follow-up 2D-TTE revealed EF of 36-40% with the development of a contained LV free wall rupture with the formation of large (8 cm) LV pseudoaneurysm outside lateral wall and multiple sites of rupture (necks) communicating with pseudoaneurysm with to-and-fro flow (Figures 3-4). He was urgently hospitalized. Chest X-ray showed cardiomegaly but no new changes, ECG showed normal sinus rhythm with non-specific ST-segment and T-wave abnormalities, Cardiac Troponin-I was 0.23, while a pre-operative coronary angiography revealed patent stents and no new development of coronary stenoses. He subsequently underwent patch repair with excellent results. Repeat 2D-TTE after three months revealed a contained pseudoaneurysm.

## Discussion

Myocardial rupture after an MI is a rare event occurring in 1.8% of cases [2]. The majority of times, bleeding may be uncontrolled and lead to pericardial tamponade, causing sudden death [3]. However, sometimes, the bleeding may be confined within the adjacent pericardium, thus creating a pseudoaneurysm. A pseudoaneurysm is characterized by a narrow neck and to-and-fro flow across the neck, thus creating a murmur [4,5]. Left ventricular free wall rupture is more likely to occur secondary to increased transmural pressure as compared to septal rupture. Considering this, the incidence of left ventricular pseudoaneurysm is still extremely low, with an incidence rate of only 0.23% in patients undergoing cardiac catheterization, as reported by Csapo et al. [1]. It develops as a complication of myocardial infarction (most common), cardiac surgery, or chest trauma [6]. The mean interval between the myocardial infarction and the diagnosis is 19 months (range 2 to 80 months) [7]. Congestive heart failure, chest pain, and dyspnea are among the common presenting symptoms, but some patients may be asymptomatic [6]. They are differentiated from true aneurysms by the absence of endocardium or myocardium and a tendency to expand and rupture.

Physical examination might reveal a murmur in more than 50% of patients, while more than 95% of the patients will have electrocardiographic changes, with the most common being non-specific ST-segment changes (74%) [6]. Chest X-ray shows cardiomegaly or evidence of a left lateral wall bulge in the majority of cases [6]. Angiography is the most accurate test and reveals a narrow orifice leading to a saccular aneurysm, thus differentiating it from a true aneurysm, which has similar dimensions of the neck and the apex [6]. It is also required as a pre-operative evaluation to determine the anatomy of coronaries as well as the need for simultaneous CABG (coronary artery bypass grafting) [8]. 2D-TTE is the best initial test, but a definitive diagnosis is made in only 26% of patients [6]. Transesophageal echocardiography and cardiac magnetic resonance imaging have an accuracy of more than 75%, however, data about their use is limited [6].

Surgery is indicated as the definitive treatment of pseudoaneurysm as it has a high risk of rupture, with a mortality rate as high as 50% without surgical intervention [6,9]. However, the case series reported by Moreno et al. recommended a conservative approach as they found no evidence of fatal rupture [10]. Moreover, a systematic review by Frances et al. and a case series by Atik et al. reported surgical mortality of 23% and 20%, respectively [6,11]. Hence, the choice of conservative management versus surgical management is up for debate and depends on patient comorbidities.

## Conclusions

Left ventricular pseudoaneurysm is a rare but fatal complication of myocardial infarction. Patients may present with congestive heart failure, chest pain, or dyspnea, and extreme vigilance is required to diagnose this condition. Our patient did not present with the aforementioned symptoms; hence, a high index of suspicion must be maintained, especially in patients with a complicated hospital course. The initial diagnostic test is 2D-TTE, but the definitive test is angiography and is almost always required as a pre-operative evaluation. Urgent surgical treatment is indicated due to the risk of rupture, however, conservative management of pseudoaneurysms has been reported but requires further research.
